# Stay-green trait improves yield, quality, and feeding value of forage oats under contrasting eco-sowing systems

**DOI:** 10.3389/fpls.2025.1709486

**Published:** 2026-01-07

**Authors:** Huimin Duan, Rui Wu, Wenhu Wang, Ruifang Zhang, Zelong Hu, Guoling Liang, Wenhui Liu

**Affiliations:** 1 Academy of Animal Science and Veterinary, Qinghai University, Xining, China; 2 Qinghai Academy of Animal Science and Veterinary, Xining, China

**Keywords:** oat, stay-green, eco-sowing systems, yield, quality

## Abstract

**Introduction:**

Oat (*Avena sativa* L.) is an important food and forage crop whose yield and quality are influenced by genotype, environmental conditions, and sowing regimes. The stay-green (SG) trait, which delays leaf senescence and maintains photosynthetic capacity, can enhance yield and forage quality; however, systematic evaluation in oat remains limited.

**Methods:**

Two genotypes—QINGYAN 3 (control, CK) and its stay-green mutant (SG)—were evaluated over two consecutive years (2023–2024) at Huangzhong, Qinghai and Yuanmou, Yunnan under contrasting eco-sowing conditions. The SG mutant was developed through field-based phenotypic selection and long-term cultivation, resulting in a stable trait. Yield, nutritional composition, forage value, and stability were evaluated. Genotype, environment, and sowing regime effects were analyzed using linear mixed models (LMM), the Technique for Order Preference by Similarity to Ideal Solution (TOPSIS), and the Analytic Hierarchy Process (AHP) .

**Results:**

The SG genotype showed significantly higher biomass, grain yield, starch, protein, and fat contents than CK. It also had lower fiber fractions (ADF and NDF) and higher digestibility indicators, including dry matter intake (DMI), digestible dry matter (DDM), total digestible nutrients (TDN), relative feed value (RFV), and relative forage quality (RFQ). Environmental and inter-annual effects were pronounced: warm, low-latitude conditions favored higher yield, while cool, high-altitude conditions enhanced nutritional composition and forage quality.

**Conclusion:**

Overall, the SG genotype outperformed CK in both yield and quality, while CK exhibited slightly greater stability across environments. The SG trait combines high yield with superior quality, providing valuable germplasm for oat improvement. Selecting genotypes according to ecological zones can further enhance forage yield and quality, supporting sustainable livestock production.

## 1 Introduction

Oat (*Avena sativa* L.) is a dual-purpose cereal, widely appreciated for its adaptability and rich nutritional profile, and forms a key component of agro-pastoral systems worldwide (Wang et al., 2025). Globally, oats are cultivated on about 8–10 million hectares, producing 19–27 million tons annually. In China, they cover roughly 1.6 × 10^5^ ha, with a total output of around 0.6 million tons (Food and Agriculture Organization of the United Nations (FAO), 2025). The nutritional quality of oat forage directly affects livestock performance, as high-quality forage is essential for maintaining animal health and enhancing meat and milk production (Tang et al., 2022; Sharma et al., 2023; Ma et al., 2024). However, global climate change poses a challenge to the consistency of oat yield and quality (Shin et al., 2020; Wang et al., 2021). These challenges are particularly severe in regions with variable climatic conditions, such as the Qinghai-Tibet Plateau and the dry-hot valley of the Jinsha River. Fluctuations in forage and grain production caused by climate change make it difficult to sustainably improve local agriculture.

The stay-green (SG) trait, which delays leaf senescence and maintains photosynthetic activity (Thomas and Howarth, 2000; Zhang et al., 2019; Chibane et al., 2021), enhances stress tolerance in crops such as maize and sorghum under adverse conditions (Liu et al., 2021; Kumar et al., 2022; Ali et al., 2023; Singh et al., 2023). SG traits improve carbon assimilation and nitrogen use efficiency during drought and other abiotic stresses by extending the photosynthetic period (Kebede et al., 2023a; b; Zhang et al., 2025). This helps mitigate the negative impacts of environmental stresses on crop yield. Similarly, a functional stay-green rice line (SNU-SG1) maintained higher chlorophyll and nitrogen levels during grain filling through sustained root activity, leading to improved grain filling and yield potential (Fu et al., 2009). However, research on the stay-green trait in oats remains limited and less comprehensive compared with that in other cereal crops.

Although oat yield and quality have been widely investigated, most previous studies have concentrated on individual traits rather than adopting an integrated framework that simultaneously considers yield, nutrient composition, feeding quality and stability (Zhang et al., 2024a, 2025). This gap is particularly evident in autumn-sown regions of Southwest China, where the lack of drought-adapted grain–forage varieties and comprehensive multi-trait evaluation systems constrains the effective selection and regional deployment of superior germplasm (Klevenhusen and Zebeli, 2021; Bryan et al., 2025).

A practical and systematic assessment of oat forage quality has been based on the combined evaluation of nutrient content and utilization efficiency, typically using parameters such as dry matter intake (DMI), digestible dry matter (DDM), total digestible nutrients (TDN), and composite indices including relative feed value (RFV) and relative feed quality (RFQ) (Rohweder et al., 1978; Moore and Undersander, 2002; Zhang et al., 2023; Fukagawa et al., 2024; Pandey et al., 2025). Incorporation of these indices into multi-trait analytical frameworks has improved the applicability of forage quality assessment and provided a scientific basis for germplasm improvement and regional adaptation studies.

Conventional evaluation approaches often focus on individual traits. This narrow focus overlooks the need for a holistic assessment that integrates yield, nutritional quality, forage value, and trait stability (Greveniotis et al., 2021; Demelash, 2024; Sokolović et al., 2024). Consequently, there is a lack of systematic frameworks capable of evaluating multiple traits simultaneously, which limits the effective selection and deployment of superior oat germplasm. Modern multi-objective evaluation frameworks, combining statistical modeling and decision analysis, allow researchers to assess multiple traits across diverse environments, providing a more complete understanding of forage performance. Traditional tools, for example the coefficient of variation, ecological valence, and genotype–environment interaction plots, are widely used to characterize the performance and stability of trial materials (Hossain et al., 2023; Demelash, 2024). Recently, approaches such as entropy weighting, expert judgment, and multi-criteria decision-making methods—including the Technique for Order Preference by Similarity to Ideal Solution (TOPSIS) and the Analytic Hierarchy Process (AHP)—have provided means to balance multiple objectives within a single framework. These integrated approaches enhance both the scientific rigor of evaluation and the practical applicability of the results for breeding and management decisions (Biswas et al., 2024; Sathiyamurthi et al., 2024).

To tackle these challenges, this study focuses on systematic multi-environment and multi-trait evaluations of spring- and autumn-sown oat ecotypes. The objectives of this study are to: (1) clarify the contribution of the stay-green trait to oat performance, (2) identify practical strategies for managing forage production in plateau and subtropical regions, and (3) establish a robust framework for comprehensive evaluation of oat germplasm. Through this approach, we aim to address the limitations of conventional single-trait assessments and provide actionable insights for oat breeding and forage management.

## 2 Materials and methods

### 2.1 Experimental sites

Field experiments were conducted in Huangzhong District (Xining, Qinghai Province) and Yuanmou County (Chuxiong Yi Autonomous Prefecture, Yunnan Province), China. Huangzhong District (101°09’–101°54’ E, 36°13’–37°03’ N) is situated at an average altitude of 2, 592 m and features a plateau continental climate. The mean annual temperature is 5.1°C, annual precipitation of 510 mm (mainly from July to September), and annual evaporation of 1, 830 mm. Yuanmou County (101°35’–102°06’ E, 25°23’–26°06’ N) lies at 1, 845 m above sea level, characterized by a dry hot subtropical monsoon climate (Subtropical dry-hot valley climate), with a mean annual temperature of 21.5°C, precipitation of 642 mm (mainly from May to October), and evaporation of 3, 627 mm.

### 2.2 Experimental design and field management

Two oat genotypes were evaluated: the control cultivar QINGYAN No.3 (CK) and its stay-green mutant QINGYAN No.3 SG (SG), both provided by the Qinghai Academy of Animal and Veterinary Sciences. QINGYAN No.3, a late-maturing dual-purpose cultivar, is officially registered and widely cultivated on the Qinghai-Tibet Plateau. The stay-green mutant was developed through field-based phenotypic selection and long-term cultivation, resulting in a stable trait.

**Figure 1:**
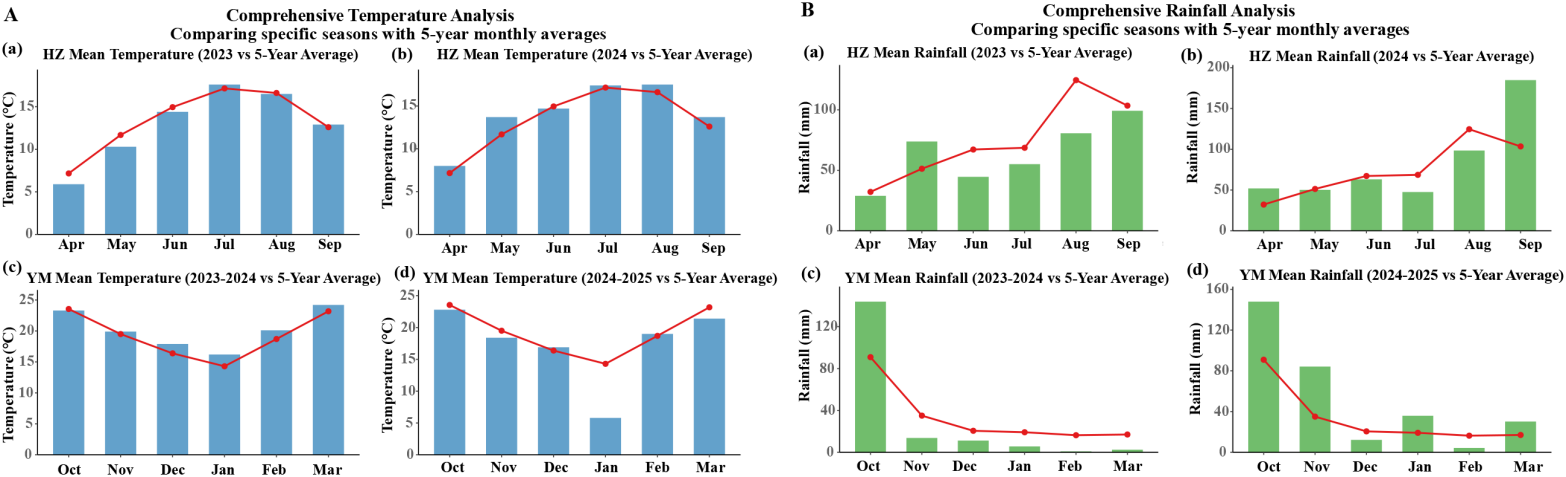
Comparison of temperature and rainfall during oat growing seasons at the experimental sites relative to the five-year local average. **(A)** Temperature; **(B)** rainfall.

A two-year, two-location experimental design was implemented. Spring sowing in Huangzhong District occurred in April 2023 and 2024, with harvest in September each year. Autumn sowing in Yuanmou County was conducted in October 2023 and 2024, with harvest in March of the following year. The climatic data for the growing season at both sites are presented in **Figure 1**.

The trials followed a randomized complete block design. Within each block, the two oat genotypes (CK and SG) were randomly assigned to plots. This randomization procedure was repeated independently for each site and year to minimize positional bias. Each genotype was planted in three replicate plots per site, each plot 15 m² (3 m × 5 m). Rows were spaced 30 cm apart, with 1 m between plots. Basal fertilization consisted of 150 kg/ha diammonium phosphate and 75 kg/ha urea; no additional fertilization was applied. Plots relied on natural precipitation, and weeds were removed manually without herbicide application. Planting density was 6.75 × 10^6^ plants/ha.

### 2.3 Plant sampling and measurements

At the physiological maturity stage of grain, all plants in each plot were harvested, leaving a 5 cm stubble. Yield-related traits were recorded as follows: single-plant fresh weight (FW; stems, leaves, leaf sheaths), single-plant dry weight (DW; after oven-drying), plot-level fresh forage yield (FY) and dry forage yield (DY), single-plant grain weight (SGW), and plot-level grain yield (GY). Grain traits, including grain length (GL), grain width (GW), grain thickness (GT), and thousand-grain weight (TGW), were measured using a digital caliper and electronic balance. After yield measurements, forage samples were heated at 105°C to inactivate enzymes, then oven-dried at 65°C, ground, and sieved through a 40-mesh screen for nutritional analysis.

#### 2.3.1 Forage nutritional composition

Dried and sieved oat samples were analyzed using near-infrared spectroscopy (NIRS) with a FOSS™ DS2500 Feed and Forage Analyser (FOSS, 2017), following standard forage evaluation protocols. Spectral data (400–2500 nm) were processed using the instrument's factory calibration models for forage composition, which have been extensively validated for common feed crops. According to the FOSS DS2500 guidelines, standard errors of prediction (SEP) for major cereal feed components—including starch, water soluble carbohydrates, protein, fat, acid detergent fiber (ADF), neutral detergent fiber (NDF), and ash—are generally below 1–1.5% for ground samples, indicating high reliability of the models. Each biological replicate was scanned five times, and the mean values of the five scans were calculated; the average of three biological replicates was then used for subsequent analyses of nutritional quality. All samples were within the calibration range of the factory models, ensuring reliable prediction of nutritional composition.

#### 2.3.2 Forage feeding value assessment

Forage feeding value indices—including dry matter intake (DMI), digestible dry matter (DDM), total digestible nutrients (TDN), relative feed value (RFV), and relative forage quality (RFQ)—were calculated using formulas described by Zhang et al (Zhang et al., 2023). Neutral detergent fiber (NDF) and acid detergent fiber (ADF) were measured experimentally, whereas DMI, DDM, and TDN were calculated for each biological replicate. DMI is expressed as a percentage of body weight, and NDF, ADF, DDM, and TDN are expressed as percentages of dry matter. RFV and RFQ were then derived from these measured and calculated nutritional components.


DMI (BW %)=120÷NDF

DDM (DW %)= 88.9−0.779×ADF

TDN%=82.38−(0.7515×ADF)

RFV = DMI×DDM÷1.29

RFQ = DMI×TDN÷1.23

### 2.4 Data analysis

Data were compiled and analyzed using Microsoft Excel 2021 (Microsoft, Redmond, WA, USA) and IBM SPSS Statistics 27 (IBM, Armonk, NY, USA). Variance analyses were conducted using linear mixed-effects models (LMM), followed by multiple comparisons using Tukey's method (*p* < 0.05) and specific pairwise comparisons.

Stability of genotypes was assessed using the coefficient of variation and Wricke's ecovalence (Wi), while genotype × environment interactions were examined via interaction plots. Combined evaluations of genotype performance were conducted using the Technique for Order Preference by Similarity to Ideal Solution (TOPSIS) with entropy and expert weighting, in conjunction with Analytic Hierarchy Process (AHP), prioritizing yield, followed by quality, with stability as an important safeguard. Pairwise comparison weightings were set as: yield vs. quality = 3; yield vs. stability = 5; quality vs. stability = 3. Consistency of expert judgments was verified using the Consistency Ratio (CR), and only matrices with CR < 0.1 were considered reliable. Graphs were generated using GraphPad Prism 10.1.2.

## 3 Results

### 3.1 Multifactorial analysis of plant biomass, yield, and grain traits

**Figure 2:**
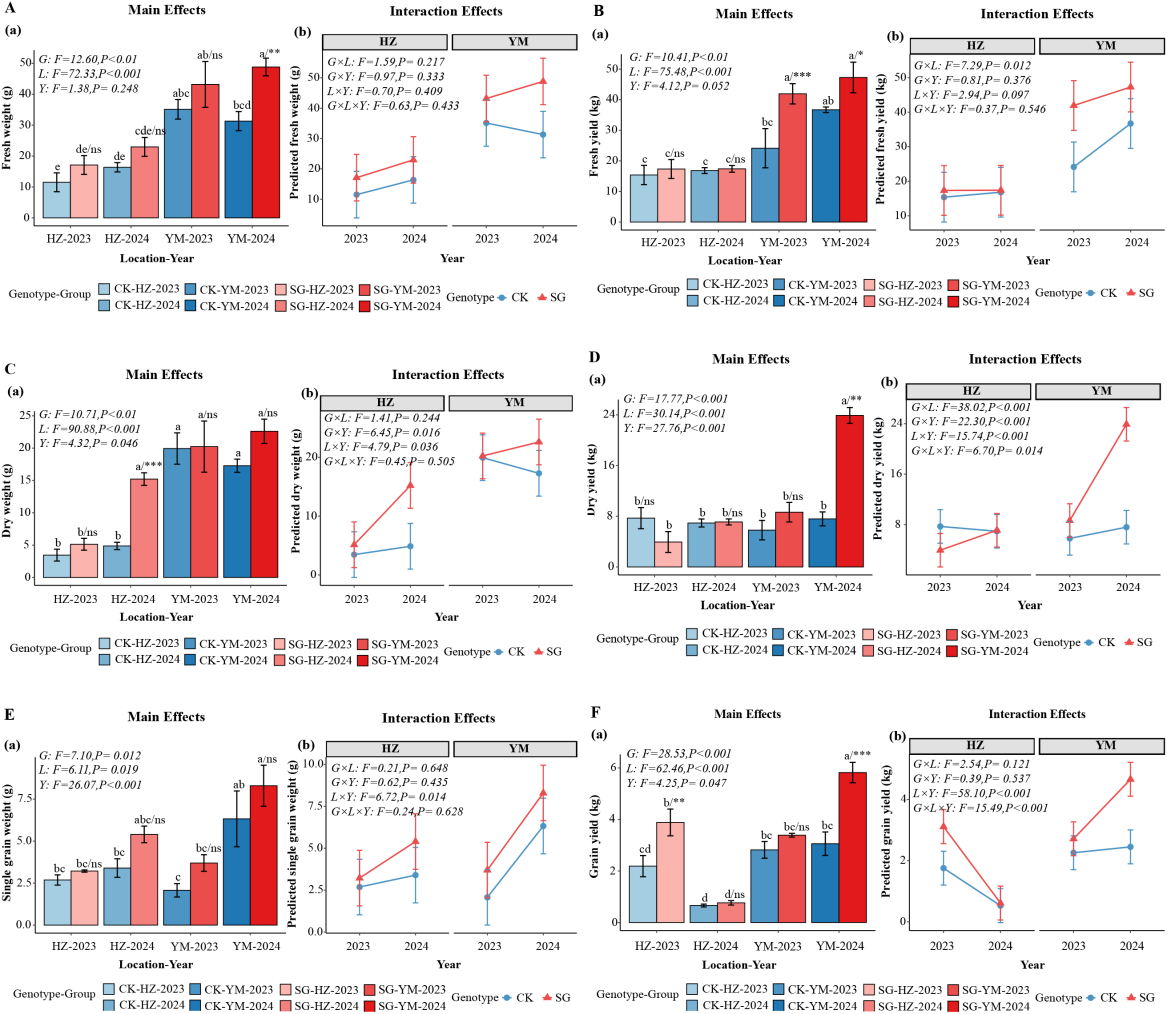
Biomass and yield performance of stay-green (SG) and control (CK) oat genotypes across two locations (HZ, YM) and two years (2023, 2024). **(A, B)** Single-plant fresh weight and fresh forage yield; **(C, D)** Single-plant dry weight and dry forage yield; **(E, F)** Single-plant and plot-level grain yield. Values are mean ± SE; G, genotype; L, location; Y, year. Statistical significance is indicated as ns (p > 0.05), * (p ≤ 0.05), ** (p ≤ 0.01), and *** (p ≤ 0.001). The different lowercase letters indicate statistically significant differences between groups at p < 0.05, as determined by Tukey’s multiple comparison test.

Across different environments and two consecutive years, stay-green (SG) oat lines consistently outperformed the control cultivar (CK) in plant biomass and yield-related traits (*p* < 0.05; **Figure 2**). Single-plant fresh weight and fresh forage yield were notably higher in SG lines, with the largest increases observed in Yuanmou (YM) and in 2024 compared with Huangzhong (HZ) and 2023, respectively (**Figures 2A, B**). Similar trends were observed for single-plant dry weight, dry forage yield, and grain yield at both the single-plant and plot levels (**Figures 2C–F**). Grain morphological traits, including length, width, thickness, and thousand-grain weight (TGW), also showed consistent advantages in SG lines, particularly in YM and in 2024 (**Supplementary Figure 1**). These results indicate that SG genotypes consistently outperform CK across locations and years, highlighting their potential for yield improvement.

To verify the observed differences, analyses of variance (ANOVA) were conducted for each individual environment and across combined environments, where environment was defined as the combination of location and year (e.g., YM-2023, HZ-2024). The results of single-environment ANOVA are summarized in **Supplementary Table S1**, and the combined ANOVA across environments is shown in **Supplementary Table S2**. Most yield and biomass-related traits exhibited significant genotype effects (*p* < 0.05) within individual environments, which remained consistent in the pooled analysis. These findings confirm that SG lines consistently outperformed CK across environments. **Supplementary Table S3** provides the mean performance, standard error (SE), and coefficient of variation (CV) of major agronomic traits for each environment, facilitating interpretation of genotype and environmental effects.

A three-way ANOVA was performed to evaluate the effects of genotype (G), location (L), and year (Y) on yield and grain-related traits. Genotype and location were the primary factors significantly affecting single-plant fresh weight and fresh forage yield (**Figures 2A, B**), single-plant dry weight and dry forage yield (**Figures 2C, D**), and single-plant and plot-level grain yield (**Figure 2E, F**) (*p* < 0.05). Dry forage yield was additionally influenced by all two-way interactions (G×L, G×Y, L×Y) and the three-way interaction (G×L×Y), whereas other yield traits showed no significant interactions. Regarding grain morphology, including grain length, width, thickness, and thousand-grain weight (TGW) (**Supplementary Figure 1**), grain length was significantly affected by genotype, location, and year; grain width was primarily influenced by location; and grain thickness was affected by location and the G×L interaction. These results highlight the dominant role of genotype and location in determining yield traits, with environmental interactions being trait-specific. The significant main effects and interactions are summarized in **Supplementary Tables S1**-**S2**, and the mean performance, SE, and CV for each environment are provided in **Supplementary Table S3** to facilitate interpretation.

### 3.2 Forage nutritional component analysis

**Figure 3:**
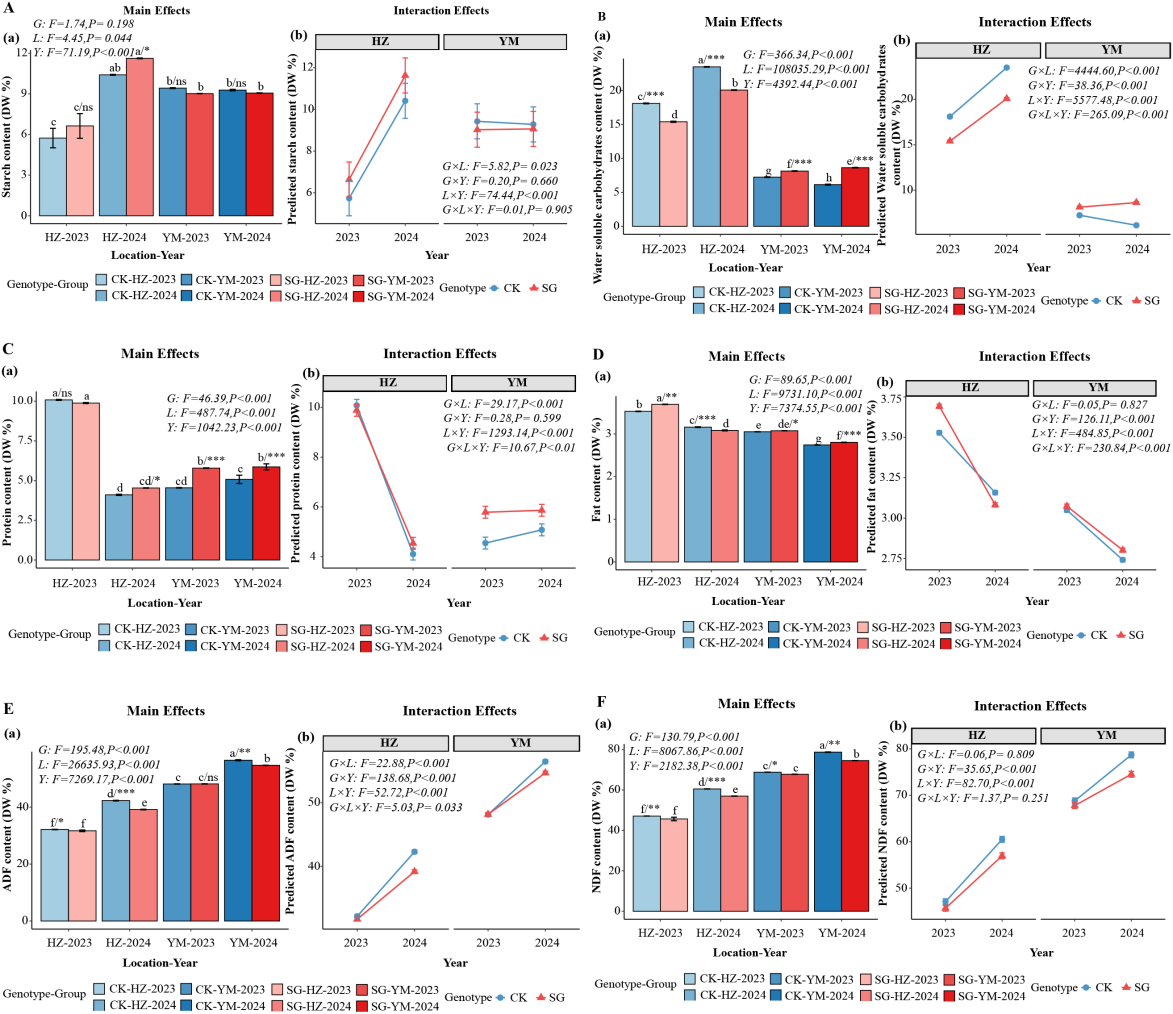
Nutritional components and fiber composition performance of stay-green (SG) and control (CK) oat genotypes across ecological zones and years. **(A-D)** Starch, water-soluble carbohydrates (WSC), protein, and fat; **(E, F)** acid detergent fiber (ADF) and neutral detergent fiber (NDF). Statistical significance is indicated as ns (p > 0.05), * (p ≤ 0.05), ** (p ≤ 0.01), and *** (p ≤ 0.001). The different lowercase letters indicate statistically significant differences between groups at p < 0.05, as determined by Tukey’s multiple comparison test.

SG oat lines consistently exhibited higher levels of major forage nutritional components compared with the control cultivar (CK) across locations and years (*p* < 0.05; **Figure 3**, **Supplementary Figure 2**). Starch, protein, and fat contents were generally higher in SG lines, while water-soluble carbohydrates (WSC) showed location and year-specific differences (**Figures 3A–D**). SG lines also had lower acid detergent fiber (ADF) and neutral detergent fiber (NDF) than CK (**Figures 3E, F**). Mineral and ash contents were similarly influenced by genotype and environment: SG lines showed higher ash, Na, Ca, and Mg contents than CK, with differences being more pronounced in specific locations and years (**Supplementary Figures 2A–D**).

ANOVA of forage nutritional traits across single and combined environments (**Supplementary Tables S1**, **S2**) showed that most quality indices (starch, protein, ADF, NDF, and minerals) were significantly affected by genotype and environment (*p* < 0.05), confirming the consistent nutritional advantage of SG lines. Summary statistics (mean, SE, and CV) are provided in **Supplementary Table S3**.

A three-way ANOVA further revealed the following patterns (*p* < 0.05): Starch content (**Figure 3A**) was significantly influenced by location, year, and the G×L and L×Y interactions. Water-soluble carbohydrates (WSC, **Figure 3B**) and fiber contents (ADF, **Figure 3E**; NDF, **Figure 3F**) were significantly affected by all main factors (G, L, Y) and interaction terms, although NDF was not influenced by G×L or G×L×Y. Protein (**Figure 3C**) and fat (**Figure 3D**) contents were primarily influenced by the main factors, with some significant interactions. Ash and mineral elements (**Supplementary Figure 2**) were significantly affected by all main factors and interactions. These results highlight the dominant role of genotype and environment in determining forage quality, with trait-specific interactions contributing to variation among locations and years.

### 3.3 Evaluation of forage feeding quality

**Figure 4:**
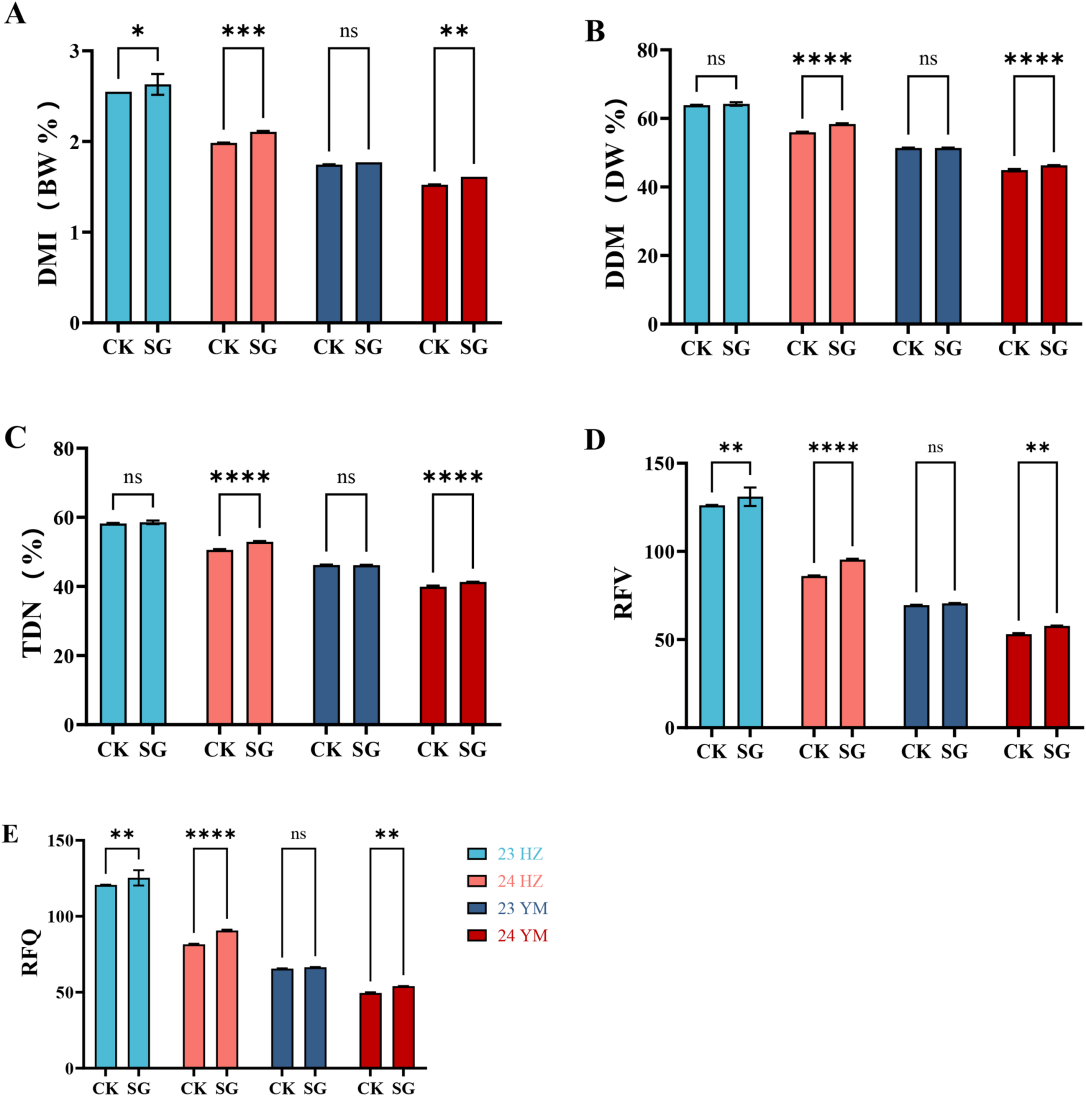
Forage feeding quality of stay-green (SG) and control (CK) oat genotypes across two locations (HZ, YM) and two years (2023, 2024). **(A-E)** Dry matter intake (DMI), digestible dry matter (DDM), total digestible nutrients (TDN), relative feed value (RFV), and relative forage quality (RFQ). Statistical significance is indicated as * (p ≤ 0.05), ** (p ≤ 0.01), *** (p ≤ 0.001), and **** (p ≤ 0.0001).

Stay-green (SG) oat lines consistently exhibited higher forage feeding quality compared with the control cultivar (CK) across locations and years (**Figure 4**). Specifically, dry matter intake (DMI, **Figure 4A**), digestible dry matter (DDM, **Figure 4B**), total digestible nutrients (TDN, **Figure 4C**), relative feed value (RFV, **Figure 4D**), and relative forage quality (RFQ, **Figure 4E**) were all higher in SG lines. Environmental effects were also evident: HZ generally showed higher values than YM, and 2023 exhibited higher values than 2024.

These results indicate that SG lines maintain superior forage feeding characteristics across ecological zones and years, with both genotype and environment contributing to variation in feeding quality.

### 3.4 Adaptability and stability analysis

**Figure 5:**
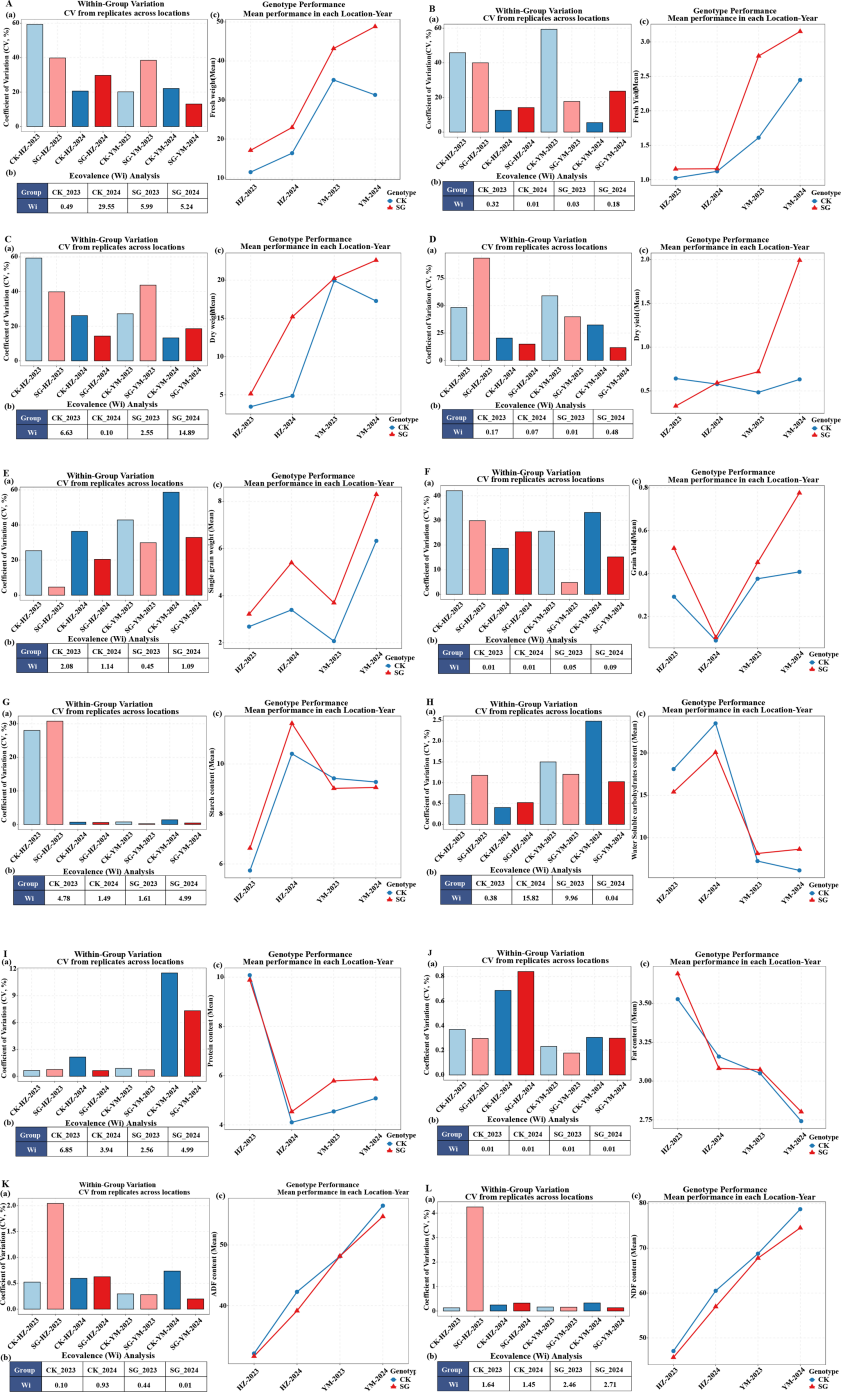
Stability and adaptability of 12 agronomic and nutritional traits for SG and CK genotypes across two locations (HZ, YM) and two years (2023, 2024). Panels **(A–L)** correspond to: **(A)** single-plant fresh weight, **(B)** fresh forage yield, **(C)** single-plant dry weight, **(D)** dry forage yield, **(E)** single-plant grain yield, **(F)** plot-level grain yield, **(G)** starch, **(H)** water-soluble carbohydrates (WSC), **(I)** protein, **(J)** fat, **(K)** acid detergent fiber (ADF), **(L)** neutral detergent fiber (NDF). Each panel includes three parts: **(a)** CV (%) across Genotype–Location–Year groups, indicating within-group consistency; **(b)** Wricke’s ecovalence (Wi) for each Genotype–Year combination, with smaller Wi values indicating higher stability; **(c)** mean performance of SG and CK across four environments, reflecting overall adaptability to environmental variation.

Stability of yield, grain, and nutritional traits was evaluated using the coefficient of variation (CV) and Wricke’s ecovalence (Wi), with genotype × environment (G×E) interaction plots used to assess adaptability (**Figure 5**, **Supplementary Figure 3**).

For yield traits, SG lines generally exhibited higher and more stable performance across environments. SG-YM-2024 showed the lowest CV values for single-plant fresh weight (13.08) and plot-level dry forage yield (11.70), while CK-YM-2024 maintained the highest stability for single-plant dry weight (13.23) and fresh forage yield (5.46). SG-HZ-2023 and SG-YM-2023 were the most stable for single-plant and plot-level grain yield, respectively.

For grain traits, stability varied among environments, with CK-HZ-2024 being most stable for grain length (CV = 2.77), SG-HZ-2024 for grain width (1.73), and CK-HZ-2023 for grain thickness (1.44) and TGW (2.48). For nutritional traits, SG lines tended to show greater stability than CK across starch, WSC, protein, fat, ADF, and NDF (CV < 15), although genotype-specific advantages were observed in certain environments. Mineral traits exhibited genotype and environment–specific patterns, with SG-YM-2024 being most stable for ash, Na, and Mg, and CK-YM-2023 for Ca.

Wi analysis revealed trends largely consistent with CV results, confirming the robustness of these patterns. Adaptability analysis further indicated significant G×E interactions (**Figure 5**), with SG lines outperforming CK in most yield traits across environments except under four specific trait–environment combinations (fresh weight in HZ-2024, single-plant fresh weight in YM-2023, dry forage yield in HZ-2023, and grain yield in HZ-2024). CK maintained relatively high stability for dry forage yield across environments, while SG lines demonstrated broader adaptability, particularly in grain morphology and nutritional quality traits.

Overall, these results indicate that stay-green genotypes combined superior yield potential with greater adaptability across diverse environments.

### 3.5 Comprehensive evaluation using TOPSIS

**Figure 6:**
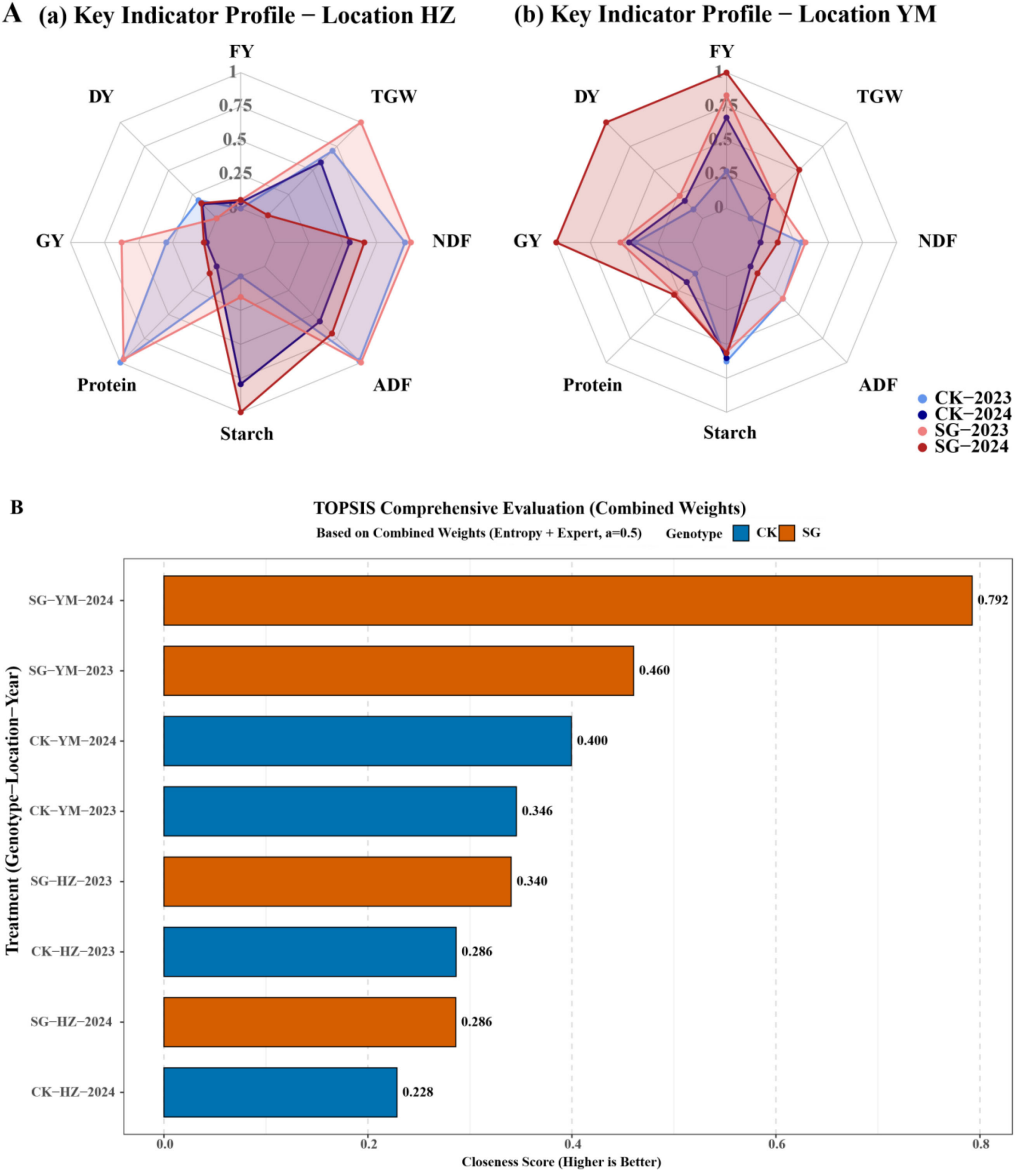
Comprehensive evaluation of SG and CK oat genotypes across two locations (HZ, YM) and two years (2023, 2024) using the TOPSIS method. **(A)** Performance of key agronomic and nutritional traits, including TGW, starch, protein, grain yield, fresh forage yield, and dry forage yield. **(B)** TOPSIS-based comprehensive ranking of genotypes across combined genotype × location × year treatments.

In HZ, SG lines exhibited superior performance in key agronomic and nutritional traits, including thousand-grain weight (TGW), starch, protein, and grain yield (**Figure 6A(a)**). In YM, SG lines showed higher fresh forage yield, TGW, grain yield, and hay yield (**Figure 6A(b)**). Based on the combined genotype × location × year analysis using the TOPSIS method, SG lines planted in YM in 2023 and 2024 achieved the highest comprehensive rankings (**Figure 6B**).

The TOPSIS (Technique for Order Preference by Similarity to Ideal Solution) method, widely used in multi-trait evaluation of crop performance, integrates multiple agronomic and nutritional indicators to generate an overall ranking of genotypes (Sathiyamurthi et al., 2024; Yu et al., 2024). This approach allows identification of genotypes with balanced performance across environments and traits. The results indicate that SG lines consistently demonstrate superior overall performance compared with CK, particularly under YM conditions, highlighting their potential for both yield and forage quality improvement.

### 3.6 AHP-based comprehensive evaluation

**Figure 7:**
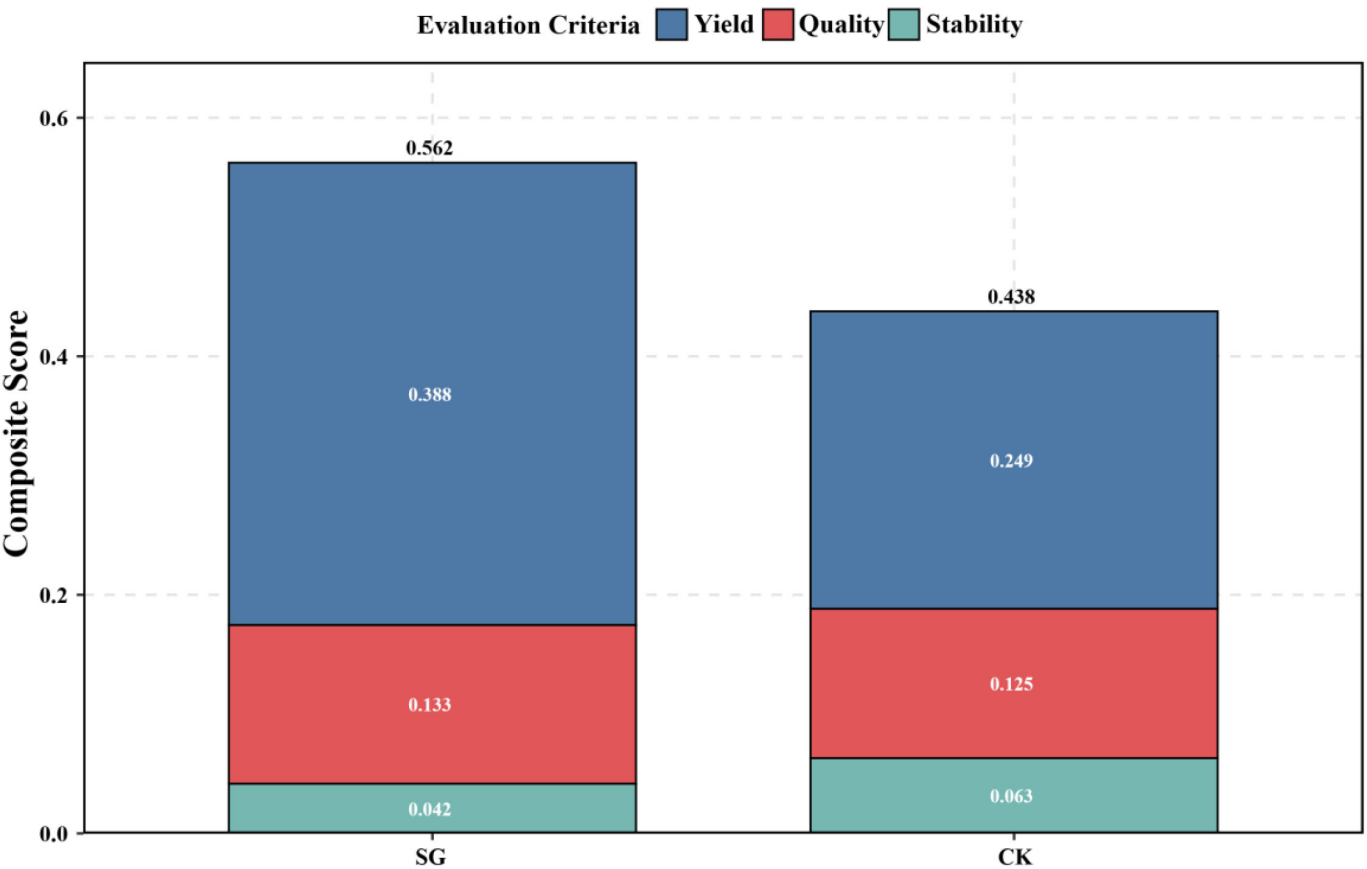
AHP-based comprehensive evaluation of oat genotypes and contributions of individual criteria.

To further conduct a systematic and quantitative evaluation of the different oat genotypes, the Analytic Hierarchy Process (AHP) was applied based on the TOPSIS model (**Figure 7**). The results indicate that SG lines achieved a higher overall AHP-based score than CK (**Figure 7A**), reflecting superior comprehensive performance across yield, quality, and stability. Yield was the primary contributor to the SG score, followed by quality and stability (**Figure 7B**). In contrast, CK exhibited lower contributions from yield and quality, but slightly higher stability compared with SG.

These findings demonstrate that, although CK showed a modest advantage in stability, SG lines are characterized by high yield and quality, resulting in superior overall performance according to the AHP-based evaluation.

## 4 Discussion

### 4.1 Yield–quality trade-offs and advantages of stay-green oats relative to conventional genotypes

Across environments, stay-green (SG) genotypes consistently outperformed conventional controls (CK) in key yield traits, including single-plant fresh weight, single-plant dry weight, and grain yield (**Figure 2**). The SG trait likely enhances yield by delaying leaf senescence, maintaining photosynthetic activity, and facilitating carbon assimilation, thereby promoting continued carbon assimilation and biomass accumulation. These mechanisms have been well documented in cereals such as maize, sorghum, and wheat (Thomas and Howarth, 2000; Zhang et al., 2019; Shin et al., 2020; Chibane et al., 2021; Wang et al., 2021; Ali et al., 2023; Singh et al., 2023), and our findings demonstrate that these mechanisms also operate in oats. Importantly, the yield advantage of SG lines was maintained across locations and years, reflecting broad environmental adaptability.

SG lines also exhibited superior forage quality compared with CK. Protein, starch, and fat contents were higher, while acid detergent fiber (ADF) and neutral detergent fiber (NDF) were lower, indicating improved digestibility and palatability (**Figure 3**). In addition, SG lines accumulated more mineral nutrients, particularly calcium and magnesium, which are essential for ruminant skeletal development and metabolic functions (**Supplementary Figure 2**) (Klevenhusen and Zebeli, 2021; Weiss and Hansen, 2024; Zhang et al., 2024b; Bryan et al., 2025; Ponnampalam et al., 2025). Taken together, these findings highlight the role of the SG trait in simultaneously enhancing yield and quality, thereby supporting the dual goals of modern forage production.

Despite the yield and quality advantages of SG lines, they exhibited greater sensitivity to environmental variation than CK, as reflected by higher fluctuations in yield and quality traits across years and locations (**Figures 2**-**4**; **Supplementary Figures 1**-**2**) (Hossain et al., 2023; Kebede et al., 2023a; Demelash, 2024; Sokolović et al., 2024; Duan et al., 2025). This pattern underscores an inherent trade-off: while SG genotypes deliver higher yield and quality, they are also more sensitive to environmental variation—a coexistence of advantages and constraints.

Comprehensive forage evaluation further supported these findings. SG consistently outperformed CK in key feeding indices, including dry matter intake (DMI), digestible dry matter (DDM), total digestible nutrients (TDN), relative feed value (RFV), and relative forage quality (RFQ) (**Figure 4**) (Zhang et al., 2023; Fukagawa et al., 2024; Pandey et al., 2025). However, TOPSIS and AHP-based analyses indicated that SG’s higher overall scores were primarily driven by yield and quality contributions, whereas CK scored relatively higher in stability (**Figures 6**, **7**) (Zhang et al., 2023). Together, the integrated evaluation indicates that SG lines are particularly suited to production systems prioritizing high yield and quality, whereas CK may perform better under stability-demanding conditions.

This coexistence of high-performance advantages and stability constraints has important implications for oat breeding and cultivation. The SG trait offers a promising route for developing high-yield, high-quality oat cultivars, supporting the dual objectives of modern forage production. At the same time, the relative reduction in stability highlights the need to consider environmental adaptability in breeding programs. Future strategies should focus on combining the SG trait with stress-resistance and stability-enhancing traits to achieve the integrated breeding objective of achieving high yield, superior quality, and robust performance across diverse environments.

### 4.2 Environmental drivers of yield–quality differentiation in contrasting eco-sowing systems

Environmental conditions strongly influenced oat yield and quality under the two representative eco-sowing systems—Huangzhong (cool highland spring sowing) and Yuanmou (subtropical hot–dry autumn sowing). Yield and quality differences between these systems highlight how environmental factors regulate trade-offs between yield and quality (**Figures 2**-**4**; **Supplementary Figure 2**).

Under the Yuanmou autumn-sowing system, fresh forage, dry forage, and grain yields were significantly higher than in Huangzhong, reflecting a high-yield advantage (**Figure 2**). This outcome aligns with the observation that warm regions with abundant thermal resources, longer frost-free periods, and extended photosynthetic duration promote dry matter accumulation (Singh et al., 2023; Zhang et al., 2024a, 2025). However, high temperature and drought stress may accelerate nutrient partitioning to grains, which reduces protein and soluble carbohydrate retention in vegetative tissues and ultimately limits forage quality (**Figure 3**).

In contrast, Huangzhong spring sowing produced forage with higher protein, water-soluble carbohydrate (WSC), and lipid contents, along with lower acid detergent fiber (ADF) and neutral detergent fiber (NDF), indicating improved digestibility and palatability (**Figure 3**). These effects likely result from cooler temperatures and sufficient moisture, which enhance carbon–nitrogen balance and delay lignification (Klevenhusen and Zebeli, 2021; Ma et al., 2024; Bryan et al., 2025). Hence, Huangzhong favors high-quality forage suitable for intensive livestock systems, despite lower overall yield. Differences in mineral composition further reflected the influence of local environmental conditions. Forage from Huangzhong contained higher ash and Na, whereas Yuanmou forage was richer in Ca and Mg, likely due to differences in soil nutrient availability and evapotranspiration (**Supplementary Figure 2**) (Weiss and Hansen, 2024). These patterns provide practical guidance for region-specific feed formulation.

Feeding quality indices confirmed these trends. Huangzhong outperformed Yuanmou across DMI, DDM, TDN, RFV, and RFQ (**Figure 4**), while Yuanmou maintained higher forage yield to meet large-scale supply demands. Interannual variation further emphasized trade-offs: yields were generally higher in 2024, whereas protein and lipid contents were greater in 2023 (**Figures 2**-**4**). Warm and humid years favor yield enhancement, whereas cooler or stress-prone years promote quality improvement (Tang et al., 2022; Sharma et al., 2023).

Overall, the two eco-sowing systems illustrate complementary production strategies rather than competing alternatives: Yuanmou favors quantity, while Huangzhong prioritizes quality. These environmental effects reinforce the classical trade-off between yield and quality and provide a basis for precise, region-specific deployment of oat genotypes.

### 4.3 Integrated advantages of the stay-green trait across eco-sowing systems

Building on the previous analysis of trade-offs between yield, quality, and stability, When data were integrated across eco-sowing systems, SG consistently showed multidimensional advantages. Under the Yuanmou system, SG achieved higher fresh forage, dry forage, and grain yields than CK, highlighting its value in quantity-oriented systems (**Figure 2**). Conversely, under the Huangzhong system, SG showed superior protein, water soluble carbohydrate, and fat contents, along with higher feeding quality indices (DMI, DDM, TDN, RFV, RFQ) (**Figure 3**-**4**), demonstrating enhanced quality under cool conditions. In other words, SG capitalizes on environmental characteristics to optimize both yield and quality.

This cross-environment performance indicates that SG is broadly adaptable, an important trait for oats serving dual-purpose roles in both grain and forage production. Multi-indicator evaluations using TOPSIS and AHP further confirmed SG’s superior scores over CK, with yield and quality advantages outweighing minor stability limitations (**Figures 6**, **7**).

Regionally targeted deployment can maximize these advantages: subtropical warm regions can exploit SG’s yield potential to expand forage supply, while highland cool regions can leverage its quality advantage for dairy and beef systems requiring high nutritional value. This flexible adaptability underscores the strategic significance of SG in future oat breeding and dissemination.

### 4.4 Outlook

This study demonstrated the integrated advantages of SG genotypes across contrasting eco-sowing systems. Future research should focus on three aspects. First, multi-site and multi-year trials are needed to separate the effects of sowing time and environment. Second, the genetic and physiological bases of SG, including carbon–nitrogen metabolism, senescence regulation, and stress responses, should be further explored using genomic and transcriptomic approaches. Third, region-specific deployment strategies should exploit SG’s yield advantage in warm environments and quality advantage in cooler regions.

## 5 Conclusion

This study systematically evaluated stay-green (SG) genotypes and conventional controls (CK) under two ecological sowing systems: spring sowing in Huangzhong and autumn sowing in Yuanmou. SG consistently outperformed CK in key yield traits (fresh weight, dry weight, grain yield), nutritional quality (protein, starch, fat contents, ADF, NDF), and feeding value indices (DMI, DDM, TDN, RFV, RFQ), demonstrating combined advantages of high yield and superior quality. Environmental conditions strongly influenced oat performance: autumn sowing in Yuanmou favored higher yield, while spring sowing in Huangzhong enhanced forage quality and feeding value, forming a complementary pattern of “high quantity” and “high quality.” Multi-indicator evaluations (TOPSIS and AHP) confirmed that SG’s comprehensive superiority was mainly driven by yield and quality, whereas CK showed slightly higher stability, reflecting a yield–stability trade-off.

Overall, the SG trait substantially enhances oats integrated production capacity across environments. These findings provide a solid theoretical and empirical basis for precision deployment of oat genotypes, region-specific cultivation, and targeted breeding strategies. In addition, the integrative framework combining physiological traits, environmental adaptation, and decision-making models (TOPSIS–AHP) offers valuable references for future studies on genotype evaluation and multi-environment optimization. Collectively, this work supports sustainable oat production systems aiming for high yield, superior quality, and environmental resilience.

## Data Availability

The raw data supporting the conclusions of this article will be made available by the authors, without undue reservation.

## Supplementary material

Supplementary Figure 1 Grain morphological traits of SG and CK oat genotypes across ecological zones and years.

Supplementary Figure 2 Mineral composition of SG and CK oat genotypes across ecological zones and years.

Supplementary Figure 3 Stability and adaptability analysis of grain morphological and mineral traits for SG and CK genotypes.

Supplementary Table 1 Analysis of variance (ANOVA) for each environment.

Supplementary Table 2 Combined ANOVA across environments.

Supplementary Table 3 Mean performance, standard error (SE), and coefficient of variation (CV) of major agronomic and quality traits across different environments.
