# Supplementary material for: Stay-green trait improves yield, quality, and feeding value of forage oats under contrasting eco-sowing systems
Source: Front Plant Sci. 2026 Jan 7;16:1709486. doi: 10.3389/fpls.2025.1709486 (PMC12819303; doi:10.3389/fpls.2025.1709486)
Supplement: Supplementary Figure 1 — Grain morphological traits of SG and CK oat genotypes across ecological zones and years. [file DataSheet1.pdf]

Supplementary Material

1.1 Supplementary Figures

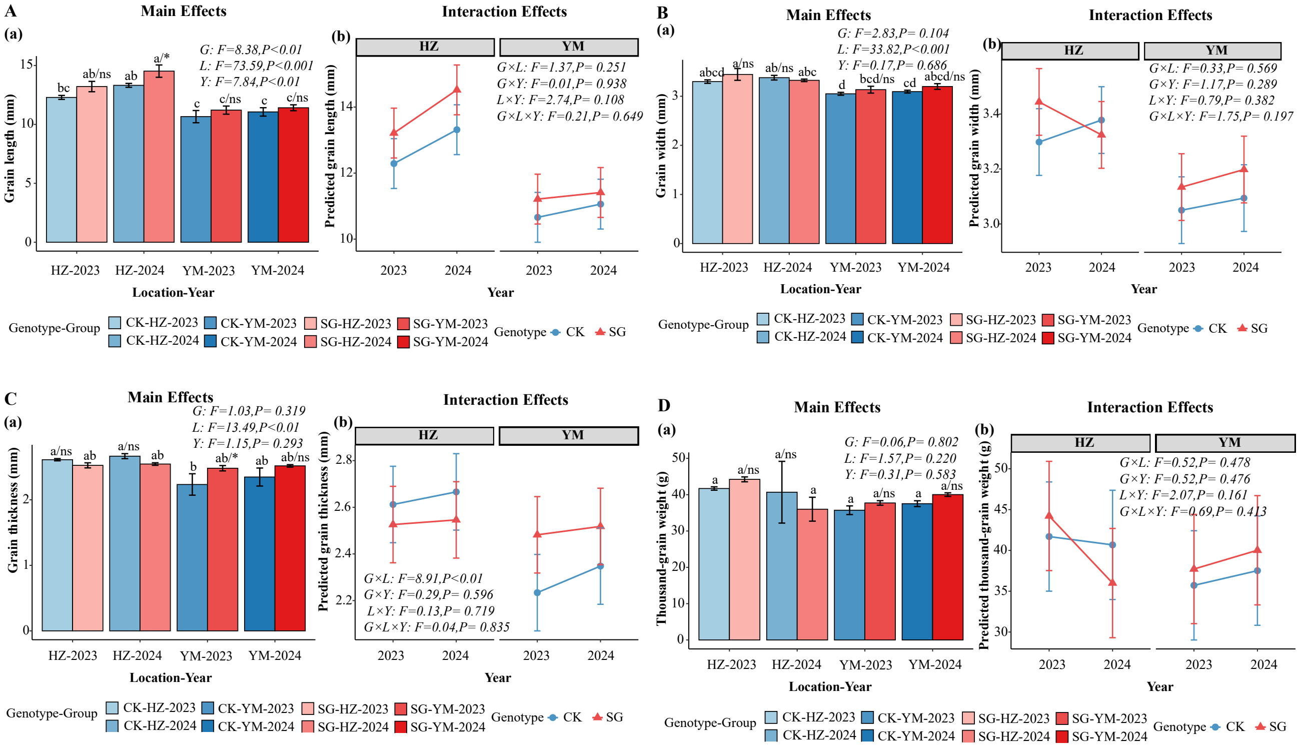

Supplementary Figure 1. Grain morphological traits of SG and CK oat genotypes across ecological zones and years. (A–D) Grain length, grain width, grain thickness, and thousand-grain weight (TGW).

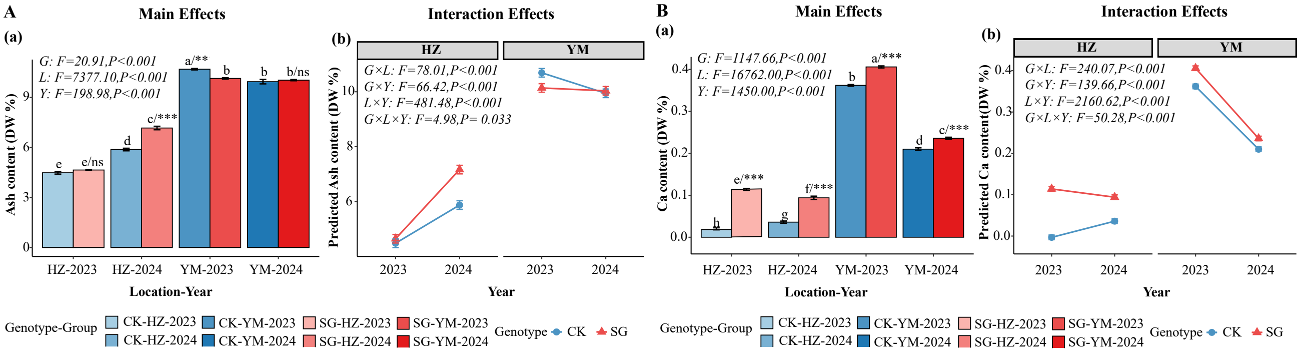

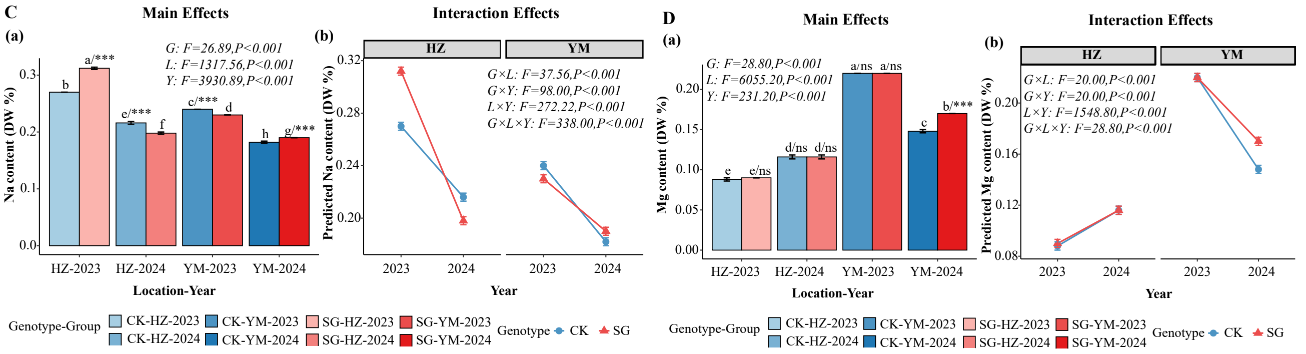

Supplementary Figure 2. Mineral composition of SG and CK oat genotypes across ecological zones and years. (A–D) Crude ash content, calcium (Ca) concentration, sodium (Na) concentration, and magnesium (Mg) concentration.

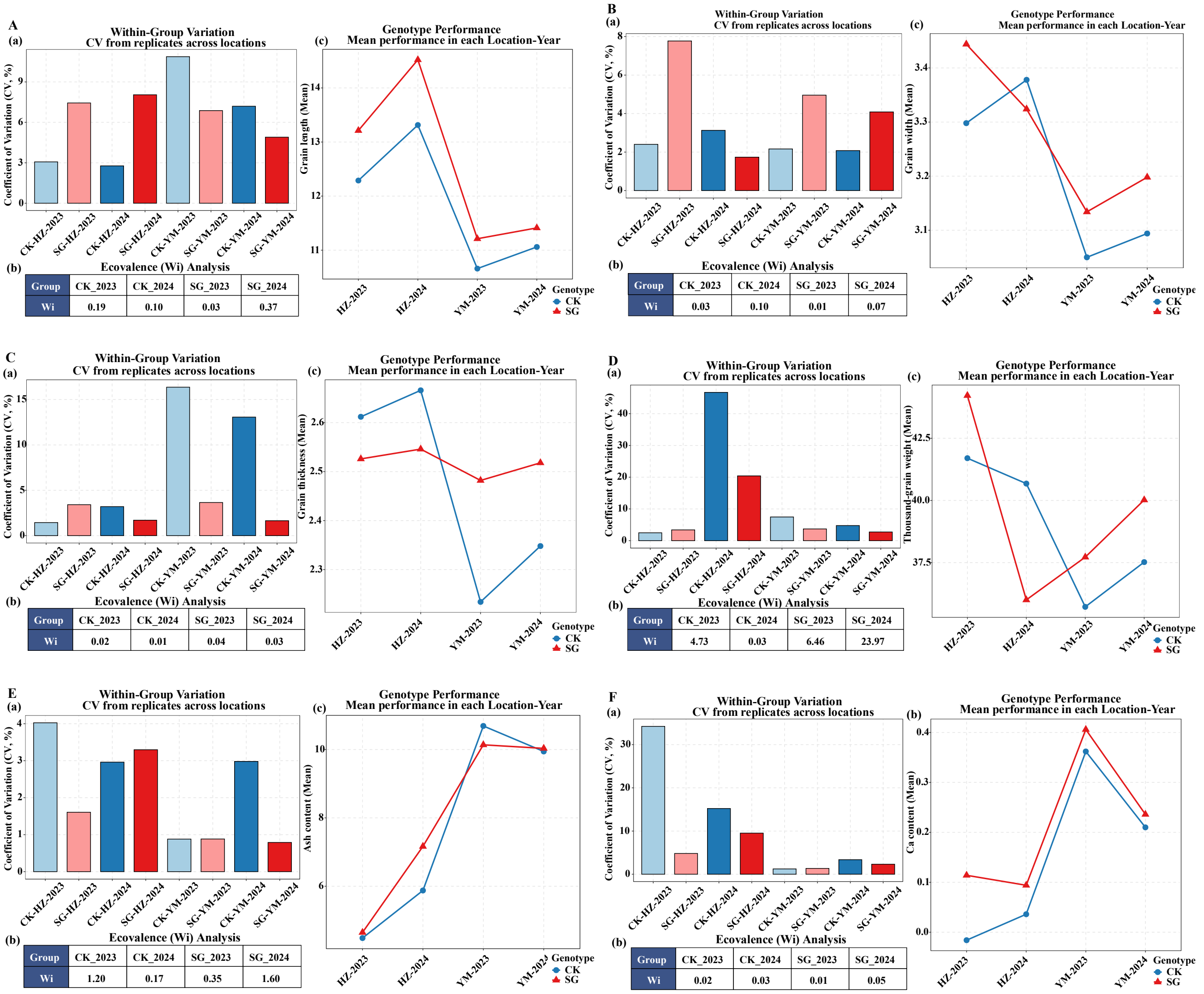

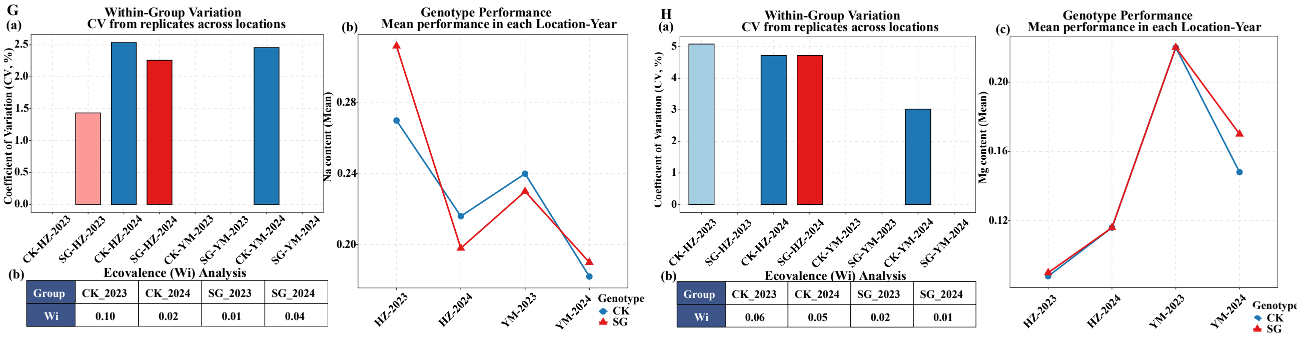

Supplementary Figure 3. Stability and adaptability of grain morphological and mineral traits for SG and CK genotypes across locations and years. Panels (A–H) correspond to: (A) grain length, (B) grain width, (C) grain thickness, (D) thousand-grain weight (TGW), (E) crude ash content, (F) calcium (Ca), (G) sodium (Na), (H) magnesium (Mg). Each panel includes three parts: (a) CV (%) across Genotype–Location–Year groups; (b) Wricke's ecovalence (Wi) for each Genotype–Year combination; (c) mean performance across four environments, illustrating adaptability.

1.2 Supplementary Tables

Supplementary Table S1. Analysis of variance (ANOVA) for each environment

Trait	Environment	Source of variation	df	Mean square	*F* value	Significance
Single-plant fresh weight（g）	HZ_2023	Genotype	1	78.18	6.01	ns
		Rep	4	80.10	6.16	ns
		Residuals	4	13.00		
	HZ_2024	Genotype	1	108.64	3.98	ns
		Rep	4	30.65	1.12	ns
		Residuals	4	27.29		
	YM_2023	Genotype	1	162.49	1.10	ns
		Rep	4	178.51	1.21	ns
		Residuals	4	147.81		
	YM_2024	Genotype	1	767.20	27.66	**

		Rep	4	60.91	2.20	ns
		Residuals	4	27.73		
		Genotype	1	9.51	0.34	ns
	HZ_2023	Rep	4	70.06	2.52	ns
		Residuals	4	27.80		
		Genotype	1	0.81	0.09	ns
	HZ_2024	Rep	4	0.95	0.10	ns
Fresh forage yield （Kg）		Residuals	4	9.60		
		Genotype	1	791.21	13.39	*
	YM_2023	Rep	4	201.50	3.41	ns
		Residuals	4	59.09		
		Genotype	1	278.78	3.28	ns
	YM_2024	Rep	4	43.79	0.52	ns
		Residuals	4	85.09		
		Genotype	1	7.04	6.01	ns
	HZ_2023	Rep	4	7.22	6.16	ns
Single-plant dry weight（g）		Residuals	4	1.17		
		Genotype	1	267.39	76.69	***
	HZ_2024	Rep	4	2.87	0.82	ns
		Residuals	4	3.49		

Dry forage yield（Kg）	YM_2023	Genotype	1	0.22	0.00	ns
		Rep	4	47.95	0.80	ns
		Residuals	4	59.74		
	YM_2024	Genotype	1	70.86	7.71	ns
		Rep	4	13.66	1.49	ns
		Residuals	4	9.20		
	HZ_2023	Genotype	1	55.46	1.93	ns
		Rep	4	14.11	0.49	ns
		Residuals	4	28.80		
	HZ_2024	Genotype	1	0.11	0.03	ns
		Rep	4	0.56	0.13	ns
		Residuals	4	4.31		
	YM_2023	Genotype	1	31.33	5.97	ns
		Rep	4	31.68	6.04	ns
		Residuals	4	5.25		
	YM_2024	Genotype	1	1040.40	90.27	***
		Rep	4	10.12	0.88	ns
		Residuals	4	11.53		
Single-plant grain weight (g)	HZ_2023	Genotype	1	0.71	3.22	ns
		Rep	4	0.27	1.21	ns

		Residuals	4	0.22		
		Genotype	1	10.04	5.91	ns
	HZ_2024	Rep	4	1.04	0.62	ns
		Residuals	4	1.70		
		Genotype	1	6.61	4.89	ns
	YM_2023	Rep	4	0.66	0.49	ns
		Residuals	4	1.35		
		Genotype	1	9.72	0.94	ns
	YM_2024	Rep	4	10.92	1.06	ns
		Residuals	4	10.32		
		Genotype	1	28.73	4.84	ns
	HZ_2023	Rep	4	2.85	0.48	ns
		Residuals	4	5.94		
		Genotype	1	0.11	1.85	ns
	HZ_2024	Rep	4	0.15	2.55	ns
Grain yield（Kg）		Residuals	4	0.06		
		Genotype	1	3.25	3.16	ns
	YM_2023	Rep	4	1.17	1.14	ns
		Residuals	4	1.03		
	YM_2024	Genotype	1	76.18	69.31	**

		Rep	4	6.16	5.60	ns
		Residuals	4	1.10		
Grain length (mm)	HZ_2023	Genotype	1	2.13	2.69	ns
		Rep	4	0.32	0.40	ns
		Residuals	4	0.79		
	HZ_2024	Genotype	1	3.64	4.05	ns
		Rep	4	0.60	0.67	ns
		Residuals	4	0.90		
	YM_2023	Genotype	1	0.76	2.46	ns
		Rep	4	1.63	5.26	ns
		Residuals	4	0.31		
	YM_2024	Genotype	1	0.31	1.97	ns
		Rep	4	0.79	5.00	ns
		Residuals	4	0.16		
Grain width (mm)	HZ_2023	Genotype	1	0.05	1.00	ns
		Rep	4	0.03	0.46	ns
		Residuals	4	0.05		
	HZ_2024	Genotype	1	0.01	1.04	ns
		Rep	4	0.01	1.06	ns
		Residuals	4	0.01		

	YM_2023	Genotype	1	0.02	0.74	ns
		Rep	4	0.01	0.19	ns
		Residuals	4	0.02		
	YM_2024	Genotype	1	0.03	1.73	ns
		Rep	4	0.01	0.35	ns
		Residuals	4	0.02		
Grain thickness (mm)	HZ_2023	Genotype	1	0.02	3.86	ns
		Rep	4	0.00	0.85	ns
		Residuals	4	0.01		
	HZ_2024	Genotype	1	0.04	8.52	*
		Rep	4	0.01	1.17	ns
		Residuals	4	0.00		
	YM_2023	Genotype	1	0.15	3.27	ns
		Rep	4	0.10	2.01	ns
		Residuals	4	0.05		
	YM_2024	Genotype	1	0.07	1.81	ns
		Rep	4	0.06	1.40	ns
		Residuals	4	0.04		
Thousand-grain weight (g)	HZ_2023	Genotype	1	15.88	8.64	*
		Rep	4	1.50	0.82	ns

		Residuals	4	1.84		
		Genotype	1	54.71	0.27	ns
	HZ_2024	Rep	4	215.58	1.08	ns
		Residuals	4	199.38		
		Genotype	1	10.00	1.75	ns
	YM_2023	Rep	4	3.37	0.59	ns
		Residuals	4	5.73		
		Genotype	1	15.63	5.66	ns
	YM_2024	Rep	4	1.64	0.59	ns
		Residuals	4	2.76		
		Genotype	1	2.01	0.96	ns
	HZ_2023	Rep	4	4.67	2.24	ns
		Residuals	4	2.08		
		Genotype	1	3.70	1374.22	***
Starch (%, DW)	HZ_2024	Rep	4	0.01	2.61	ns
		Residuals	4	0.00		
		Genotype	1	0.40	146.38	***
	YM_2023	Rep	4	0.00	1.01	ns
		Residuals	4	0.00		
	YM_2024	Genotype	1	0.12	8.54	*

		Rep	4	0.00	0.32	ns
		Residuals	4	0.01		
	HZ_2023	Genotype	1	18.25	974.22	***
		Rep	4	0.03	1.66	ns
		Residuals	4	0.02		
	HZ_2024	Genotype	1	29.28	5513.22	***
		Rep	4	0.02	2.79	ns
Water soluble carbohydrates (%, DW)		Residuals	4	0.01		
	YM_2023	Genotype	1	2.00	203.58	***
		Rep	4	0.01	1.18	ns
		Residuals	4	0.01		
	YM_2024	Genotype	1	15.60	4233.38	***
		Rep	4	0.03	7.44	*
		Residuals	4	0.00		
Protein (%, DW)	HZ_2023	Genotype	1	0.10	183.20	***
		Rep	4	0.01	17.45	**
		Residuals	4	0.00		
	HZ_2024	Genotype	1	0.47	84.29	***
		Rep	4	0.00	0.56	ns

		Residuals	4	0.01		
	YM_2023	Genotype	1	3.84	1537.60	***
		Rep	4	0.00	0.34	ns
		Residuals	4	0.00		
	YM_2024	Genotype	1	1.54	4.14	ns
		Rep	4	0.16	0.42	ns
		Residuals	4	0.37		
	HZ_2023	Genotype	1	0.07	407.52	***
		Rep	4	0.00	0.76	ns
		Residuals	4	0.00		
	HZ_2024	Genotype	1	0.01	14.96	*
		Rep	4	0.00	0.18	ns
		Residuals	4	0.00		
Fat (%, DW)	YM_2023	Genotype	1	0.00	36.00	**
		Rep	4	0.00	1.00	ns
		Residuals	4	0.00		
	YM_2024	Genotype	1	0.01	90.00	***
		Rep	4	0.00	0.40	ns
		Residuals	4	0.00		
	HZ_2023	Genotype	1	0.51	4.17	ns

		Rep	4	0.33	2.65	ns
		Residuals	4	0.12		
		Genotype	1	24.37	396.47	***
	HZ_2024	Rep	4	0.06	1.01	ns
		Residuals	4	0.06		
Acid detergent fiber (%, DW)		Genotype	1	0.00	0.18	ns
	YM_2023	Rep	4	0.02	1.62	ns
		Residuals	4	0.02		
		Genotype	1	7.96	73.44	**
	YM_2024	Rep	4	0.08	0.70	ns
		Residuals	4	0.11		
		Genotype	1	5.01	2.79	ns
	HZ_2023	Rep	4	1.97	1.10	ns
		Residuals	4	1.80		
		Genotype	1	31.40	2349.41	***
Neutral detergent fiber (%, DW)	HZ_2024	Rep	4	0.04	3.19	ns
		Residuals	4	0.01		
		Genotype	1	2.53	467.24	***
	YM_2023	Rep	4	0.02	3.35	ns
		Residuals	4	0.01		

		Genotype	1	43.47	973.08	***
	YM_2024	Rep	4	0.03	0.71	ns
		Residuals	4	0.05		
		Genotype	1	0.07	4.43	ns
	HZ_2023	Rep	4	0.02	1.51	ns
		Residuals	4	0.02		
		Genotype	1	4.16	83.25	***
	HZ_2024	Rep	4	0.04	0.72	ns
		Residuals	4	0.05		
Ash (%, DW)		Genotype	1	0.76	64.37	**
	YM_2023	Rep	4	0.01	0.43	ns
		Residuals	4	0.01		
		Genotype	1	0.02	0.34	ns
	YM_2024	Rep	4	0.03	0.56	ns
		Residuals	4	0.06		
		Genotype	1	0.04	2.39E+32	***
	HZ_2023	Rep	4	0.00	3.40E+29	***
CA (%, DW)		Residuals	4	0.00		
	HZ_2024	Genotype	1	0.01	98.94	***
		Rep	4	0.00	0.29	ns

		Residuals	4	0.00		
		Genotype	1	0.01	322.67	***
	YM_2023	Rep	4	0.00	2.33	ns
		Residuals	4	0.00		
		Genotype	1	0.00	26.00	**
	YM_2024	Rep	4	0.00	0.23	ns
		Residuals	4	0.00		
		Genotype	1	0.00	441.00	***
	HZ_2023	Rep	4	0.00	1.00	ns
		Residuals	4	0.00		
		Genotype	1	0.00	23.14	**
	HZ_2024	Rep	4	0.00	0.43	ns
		Residuals	4	0.00		
NA (%, DW)		Genotype	1	0.00	5.58E+28	***
	YM_2023	Rep	4	0.00	1.00	ns
		Residuals	4	0.00		
		Genotype	1	0.00	16.00	*
	YM_2024	Rep	4	0.00	1.00	ns
		Residuals	4	0.00		
MG (%, DW)	HZ_2023	Genotype	1	0.00	1.00	ns

	Rep	4	0.00	1.00	ns
	Residuals	4	0.00		
	Genotype	1	0.00	0.00	ns
HZ_2024	Rep	4	0.00	0.20	ns
	Residuals	4	0.00		
	Genotype	1	0.00	1.00	ns
YM_2023	Rep	4	0.00	1.00	ns
	Residuals	4	0.00		
	Genotype	1	0.00	121.00	***
YM_2024	Rep	4	0.00	1.00	ns
	Residuals	4	0.00	6.01	

Supplementary Table S2. Combined ANOVA across environments

Trait	Source of variation	df	Mean square	*F* value	Significance
Single-plant fresh weight （g）	Genotype	1	891.33	12.43	**
	Environment	3	1754.99	24.47	***
	Rep	4	63.89	0.89	ns
	Genotype x Environment	3	75.06	1.05	ns
	Residuals	28	71.73		
	Genotype	1	595.60	10.41	**

Fresh forage yield（Kg）	Environment	3	1574.30	27.51	***
	Rep	4	97.33	1.70	ns
	Genotype x Environment	3	161.57	2.82	ns
	Residuals	28	57.22		
Single-plant dry weight（g）	Genotype	1	194.44	9.86	**
	Environment	3	605.33	30.69	***
	Rep	4	7.23	0.37	ns
	Genotype x Environment	3	50.36	2.55	ns
	Residuals	28	19.72		
Dry forage yield （Kg）	Genotype	1	236.20	16.85	***
	Environment	3	326.35	23.28	***
	Rep	4	8.22	0.59	ns
	Genotype x Environment	3	297.03	21.19	***
	Residuals	28	14.02		
Single-plant grain weight (g)	Genotype	1	23.52	7.10	*
	Environment	3	42.92	12.96	***
	Rep	4	3.30	1.00	ns
	Genotype x Environment	3	1.19	0.36	ns
	Residuals	28	3.31		
	Genotype	1	65.79	25.87	***

Grain yield（Kg）	Environment	3	95.93	37.72	***
	Rep	4	0.65	0.25	ns
	Genotype x Environment	3	14.16	5.57	**
	Residuals	28	2.54		
Grain length (mm)	Genotype	1	5.75	8.17	**
	Environment	3	19.26	27.35	***
	Rep	4	0.56	0.80	ns
	Genotype x Environment	3	0.36	0.52	ns
	Residuals	28	0.70		
Grain width (mm)	Genotype	1	0.05	2.83	ns
	Environment	3	0.20	11.59	***
	Rep	4	0.02	1.20	ns
	Genotype x Environment	3	0.02	1.08	ns
	Residuals	28	0.02		
Grain thickness (mm)	Genotype	1	0.03	1.03	ns
	Environment	3	0.14	4.92	**
	Rep	4	0.06	2.36	ns
	Genotype x Environment	3	0.08	3.08	*
	Residuals	28	0.03		
	Genotype	1	3.43	0.06	ns

Thousand-grain weight (g)	Environment	3	70.71	1.32	ns
	Rep	4	56.15	1.05	ns
	Genotype x Environment	3	30.93	0.58	ns
	Residuals	28	53.66		
Starch (%, DW)	Genotype	1	1.39	1.74	ns
	Environment	3	40.07	50.03	***
	Rep	4	1.18	1.48	ns
	Genotype x Environment	3	1.61	2.01	ns
	Residuals	28	0.80		
Water soluble carbohydrates (%, DW)	Genotype	1	4.67	366.34	***
	Environment	3	500.88	39335.07	***
	Rep	4	0.03	2.61	ns
	Genotype x Environment	3	20.15	1582.68	***
	Residuals	28	0.01		
Protein (%, DW)	Genotype	1	3.19	45.23	***
	Environment	3	64.75	917.50	***
	Rep	4	0.06	0.80	ns
	Genotype x Environment	3	0.92	13.04	***
	Residuals	28	0.07		
Fat (%, DW)	Genotype	1	0.02	80.39	***

	Environment	3	1.21	5258.03	***
	Rep	4	0.00	0.17	ns
	Genotype x Environment	3	0.03	106.71	***
	Residuals	28	0.00		
Acid detergent fiber (%, DW)	Genotype	1	17.73	195.48	***
	Environment	3	1026.58	11319.27	***
	Rep	4	0.16	1.76	ns
	Genotype x Environment	3	5.04	55.53	***
	Residuals	28	0.09		
Neutral detergent fiber (%, DW)	Genotype	1	64.21	129.45	***
	Environment	3	1690.98	3408.94	***
	Rep	4	0.46	0.92	ns
	Genotype x Environment	3	6.07	12.23	***
	Residuals	28	0.50		
Ash (%, DW)	Genotype	1	0.62	20.54	***
	Environment	3	78.99	2638.29	***
	Rep	4	0.03	0.86	ns
	Genotype x Environment	3	1.47	48.92	***
	Residuals	28	0.03		
CA (%, DW)	Genotype	1	0.04	1147.66	***

	Environment	3	0.25	6790.87	***
	Rep	4	0.00	1.28	ns
	Genotype x Environment	3	0.01	143.33	***
	Residuals	28	0.00		
NA (%, DW)	Genotype	1	0.00	26.06	***
	Environment	3	0.02	1783.60	***
	Rep	4	0.00	0.75	ns
	Genotype x Environment	3	0.00	153.00	***
	Residuals	28	0.00		
MG (%, DW)	Genotype	1	0.00	27.62	***
	Environment	3	0.03	2504.40	***
	Rep	4	0.00	0.67	ns
	Genotype x Environment	3	0.00	21.99	***
	Residuals	28	0.00		

Supplementary Table S3. Mean performance, standard error (SE), and coefficient of variation (CV) of major agronomic and quality traits across different environments.

Trait	Environment	Mean	SE	CV (%)
Single-plant fresh weight （g）	HZ_2023	14.31	2.24	49.43
	HZ_2024	19.67	1.95	31.27
	YM_2023	39.16	4.04	32.61

	YM_2024	40.07	3.53	27.87
Fresh forage yield（Kg）	HZ_2023	16.37	2.11	40.78
	HZ_2024	17.12	0.69	12.78
	YM_2023	33.05	4.51	43.19
	YM_2024	42.00	2.97	22.37
Single-plant dry weight （g）	HZ_2023	4.30	0.67	49.45
	HZ_2024	10.05	1.80	56.79
	YM_2023	20.09	2.19	34.44
	YM_2024	19.94	1.34	21.29
Dry forage yield （Kg）	HZ_2023	7.28	1.59	69.05
	HZ_2024	8.78	0.47	16.81
	YM_2023	9.03	1.41	49.39
	YM_2024	19.68	3.54	56.86
Single-plant grain weight (g)	HZ_2023	2.95	0.17	18.40
	HZ_2024	4.40	0.48	34.74
	YM_2023	2.88	0.40	44.26
	YM_2024	7.31	1.03	44.36
Grain yield （Kg）	HZ_2023	6.08	0.84	43.85
	HZ_2024	1.43	0.10	22.87
	YM_2023	6.21	0.37	18.61

	YM_2024	8.88	1.08	38.50
Grain length (mm)	HZ_2023	12.75	0.27	6.70
	HZ_2024	13.92	0.33	7.44
	YM_2023	10.94	0.31	8.89
	YM_2024	11.24	0.21	6.00
Grain width (mm)	HZ_2023	3.37	0.06	5.97
	HZ_2024	3.35	0.03	2.54
	YM_2023	3.09	0.04	3.91
	YM_2024	3.15	0.04	3.54
Grain thickness (mm)	HZ_2023	2.57	0.02	3.01
	HZ_2024	2.61	0.03	3.45
	YM_2023	2.36	0.09	12.01
	YM_2024	2.43	0.07	9.25
Thousand-grain weight (g)	HZ_2023	42.96	0.57	4.20
	HZ_2024	38.34	4.37	36.00
	YM_2023	36.72	0.72	6.18
	YM_2024	38.77	0.61	4.96
Starch (%, DW)	HZ_2023	6.19	0.57	29.04
	HZ_2024	11.02	0.20	5.85
	YM_2023	9.23	0.07	2.36

	YM_2024	9.17	0.05	1.59
Water soluble carbohydrates (%, DW)	HZ_2023	16.76	0.45	8.55
	HZ_2024	21.79	0.57	8.29
	YM_2023	7.69	0.15	6.26
	YM_2024	7.39	0.42	17.88
Protein (%, DW)	HZ_2023	9.98	0.04	1.24
	HZ_2024	4.32	0.08	5.46
	YM_2023	5.17	0.21	12.67
	YM_2024	5.47	0.20	11.66
Fat (%, DW)	HZ_2023	3.61	0.03	2.42
	HZ_2024	3.12	0.02	1.47
	YM_2023	3.06	0.00	0.46
	YM_2024	2.77	0.01	1.18
Acid detergent fiber (%, DW)	HZ_2023	31.92	0.16	1.59
	HZ_2024	40.73	0.53	4.08
	YM_2023	48.15	0.04	0.27
	YM_2024	55.58	0.31	1.77
Neutral detergent fiber (%, DW)	HZ_2023	46.37	0.47	3.22
	HZ_2024	58.73	0.59	3.19
	YM_2023	68.29	0.17	0.79

	YM_2024	76.62	0.70	2.88
Ash (%, DW)	HZ_2023	4.57	0.05	3.42
	HZ_2024	6.52	0.22	10.85
	YM_2023	10.41	0.10	2.92
	YM_2024	9.99	0.07	2.10
CA (%, DW)	HZ_2023	0.05	0.02	140.23
	HZ_2024	0.07	0.01	48.24
	YM_2023	0.38	0.01	6.16
	YM_2024	0.22	0.01	6.70
NA (%, DW)	HZ_2023	0.29	0.01	7.68
	HZ_2024	0.21	0.00	5.12
	YM_2023	0.24	0.00	2.24
	YM_2024	0.19	0.00	2.78
MG (%, DW)	HZ_2023	0.09	0.00	3.55
	HZ_2024	0.12	0.00	4.45
	YM_2023	0.22	0.00	0.00
	YM_2024	0.16	0.00	7.53
